# ﻿Description of a new uniformly brown estuarine moray eel (Anguilliformes, Muraenidae) from the Central Indo-Pacific Ocean

**DOI:** 10.3897/zookeys.1220.129685

**Published:** 2024-12-09

**Authors:** Wen-Chien Huang, Yusuke Hibino, Rodulf Anthony Balisco, Te-Yu Liao

**Affiliations:** 1 Department of Oceanography, National Sun Yat-sen University, Kaohsiung, Taiwan National Sun Yat-sen University Kaohsiung Taiwan; 2 Kitakyushu Museum of Natural History and Human History, Kitakyushu, Fukuoka, Japan Kitakyushu Museum of Natural History and Human History Kitakyushu Japan; 3 College of Fisheries and Aquatic Sciences, Western Philippines University, Puerto Princesa City, Philippines Western Philippines University Puerto Princesa City Philippines

**Keywords:** DNA barcoding, mangroves, unicolor snake moray, Uropterygiinae, *Uropterygiusmactanensis*

## Abstract

A new estuarine moray eel, *Uropterygiushades***sp. nov.**, is described based on 14 specimens from Japan, Taiwan, the Philippines, southern Indonesia, and Fiji. It is a small-bodied, slender, uniformly dark-brown moray separated from congeners within the *U.concolor* species complex. The new species can be distinguished from congeners by the anteriorly positioned small eyes (5.0–7.2% of head length), absence of branchial pores, and extended inner rows of teeth which reach the posterior end of the jaws. *Uropterygiushades***sp. nov.** represents a rare species of moray eel that inhabits turbid estuarine environments, preferring soft, muddy substrates, and burrowing and hiding among rocks or in fallen mangrove leaves. Additionally, *Uropterygiusmactanensis* Huang, Balisco, Evacitas & Liao, another species recently separated from the *U.concolor* species complex, is reported for the first time from Iriomote Island in the Ryukyu Archipelago based on two specimens; this new record expands the geographic range of *U.mactanensis* from the central Philippines to southern Japan.

## ﻿Introduction

**Figure 1.**
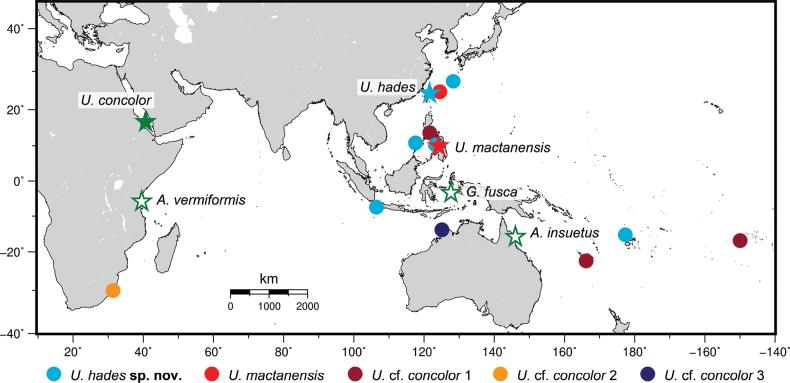
The distribution of nominal species and genetic lineages of the *Uropterygiusconcolor* species complex in the Indo-Pacific region. Each color represents a species or genetic lineage. Solid stars indicate the type localities of valid species; empty green stars indicate type localities of the three junior synonyms of *U.concolor*. Figure modified from Huang et al. (2023a).

*Uropterygius* Rüppell, 1838 is the most speciose genus of the subfamily Uropterygiinae (family Muraenidae), comprising 23 of the 38 valid species in the subfamily (Smith 2012; Huang et al. 2023a, 2023b; Fricke et al. 2024). The type species of the genus is *Uropterygiusconcolor* Rüppell, 1838, a small-bodied, uniformly brown moray eel described from Eritrea in the Red Sea. Three nominal species described outside the Red Sea have been synonymized with *U.concolor*, including *Anarchiasinsuetus* Whitley, 1932, *Anarchiasvermiformis* Smith, 1962, and *Gymnomuraenafusca* Peters, 1866 (Böhlke and Smith 2002) (Fig. 1). Consequently, *U.concolor* was considered a widespread species in the Indo-Pacific region, distributed from South Africa to the Marquesas Islands, north to southern Japan, and south to New Caledonia (McCosker et al. 1984; Fricke et al. 2011, 2018; Delrieu-Trottin et al. 2015).

However, recent molecular studies based on cytochrome c oxidase subunit I (*COI*) sequences have revealed that, in addition to the true *U.concolor*, there are at least five deeply divergent genetic lineages identified as “*U.concolor*” (Smith et al. 2019; Huang et al. 2023a). These studies suggested that the true *U.concolor* is currently known only from the Red Sea, while other genetic lineages outside the Red Sea likely represent a species complex. Among these lineages, one was recently described as *Uropterygiusmactanensis* Huang, Balisco, Evacitas & Liao, 2023 from Cebu, the Philippines, while the remaining lineages were only identified as U.cf.concolor 1 (New Caledonia and the Society Islands), U.cf.concolor 2 (South Africa), U.cf.concolor 3 (Western Australia), and U.cf.concolor 4 (Okinawa) (Smith et al. 2019; Huang et al. 2023a) (Fig. 1). Additionally, the synonymization of *Anarchiasinsuetus*, *A.vermiformis*, and *Gymnomuraenafusca* with *U.concolor* remain uncertain (Huang et al. 2023a).

Sakai and Sato (1982) provided the first brief description of U.cf.concolor 4 based on two specimens collected from estuaries of the Amami and Okinawa islands in southern Japan. However, they identified it as *U.concolor*, and this misidentification was perpetuated in all subsequent Japanese reports. Although McCosker et al. (1984) questioned the identification of Japanese specimens by noting the absence of a branchial pore compared to those from the Red Sea, they attributed this variation to different environmental conditions, as they did not find any other morphological differences. According to literature, photos, and specimen records worldwide, U.cf.concolor 4 is mainly found in estuarine mangrove swamps in the Ryukyu Archipelago, such as Amami, Okinawa, Ishigaki, and Iriomote islands (Hatooka and Yoshino 1982; Sakai and Sato 1982; McCosker et al. 1984; Maeda and Tachihara 2006; Kanda et al. 2009; Hibino 2018; Miyake et al. 2019). It has been listed as a Critically Endangered (CR) species in the Red Data Book by the Okinawa Prefectural Government due to its rarity and habitat destruction (Tachihara 2017). However, the molecular characteristics of U.cf.concolor 4 were not available until Miyake et al. (2019) sequenced the mitochondrial genome of two specimens from Okinawa Island. Subsequently, Huang et al. (2023a) used these sequences for comparison and identified diagnostic morphological characteristics for U.cf.concolor 4, suggesting that it is possibly an undescribed species. Furthermore, they examined two specimens from Fiji and southern Java that seem similar to U.cf.concolor 4 and proposed that it may be widely distributed in the central and western Pacific Ocean, in addition to its occurrence in the Ryukyu Archipelago.

In the present study, we conducted detailed examinations of the specimens initially identified as U.cf.concolor 4 and describe it as a new species based on 14 specimens from Japan, Taiwan, the Philippines, southern Indonesia, and Fiji. This new species represents a rare case of a widespread moray eel specifically inhabiting estuarine environments. Additionally, during the examination, we found two specimens from Iriomote Island that can be recognized as *U.mactanensis*. This finding represents the first record of *U.mactanensis* in this area and signifies a northward range expansion from the central Philippines to the Ryukyu Archipelago.

## ﻿Material and methods

Sixteen specimens (14 Uropterygiuscf.concolor 4 and two *U.mactanensis*) were examined, mostly from museum collections, with a few newly obtained samples. Fresh specimens were photographed, and a piece of muscle tissue was obtained from a small incision in the abdomen near the anus. Tissue samples were preserved in 95% ethanol in a −20 °C freezer prior to DNA extraction, while the voucher specimens were fixed in 10% formalin before gradually transferred to 70% ethanol for long-term preservation. Morphological data were collected from the specimens deposited in different institutions, including National Museum of Marine Biology and Aquarium, Pingtung (**NMMB-P**), Kagoshima University Museum, Kagoshima (**KAUM–I**), Kitakyushu Museum of Natural History and Human History, Kitakyushu, Fukuoka (**KMNH VR**), Kyushu University Museum, Fukuoka (**KYUM-PI**), Okinawa Churashima Foundation, Motobu, Okinawa (**OCF**; the two **URM-P** specimens have been donated to OCF), National Museum of the Philippines, Manila (**PNM**), Australian Museum, Sydney (**AMS I**), and Zoological Reference Collection, Lee Kong Chian Natural History Museum, Singapore (ZRC). Specimens from the Ryukyu Archipelago identified as “*U.concolor*” were deposited in various Japanese institutions. In addition to the previously mentioned museums, specimens from Graduate School & Faculty of Bioresources, Mie University, Tsu, Mie (**FRLM**), National Museum of Nature and Science, Tsukuba, Ibaraki (**NSMT-P**), and the University Museum, the University of Tokyo, Tokyo (**ZUMT**), were also examined and confirmed by the second author. However, due to their condition, only 11 specimens (nine U.cf.concolor 4 and two *U.mactanensis*) were selected for detailed examination.

Morphometrics were measured following Böhlke et al. (1989), and these characters are presented as percentages of total length (**TL**) or head length (**HL**). Meristic counts include vertebrae, teeth, and cephalic sensory pores. Vertebral numbers were counted from radiographs, and the vertebral formula is presented as pre-anus, pre-dorsal fin, pre-anal fin, and total vertebrae, following the definitions of Böhlke (1982) with slight modifications. Dentition and head pores were examined under a stereomicroscope, with terminologies following Böhlke and Smith (2002) and Smith et al. (2019), respectively.

**Figure 2.**
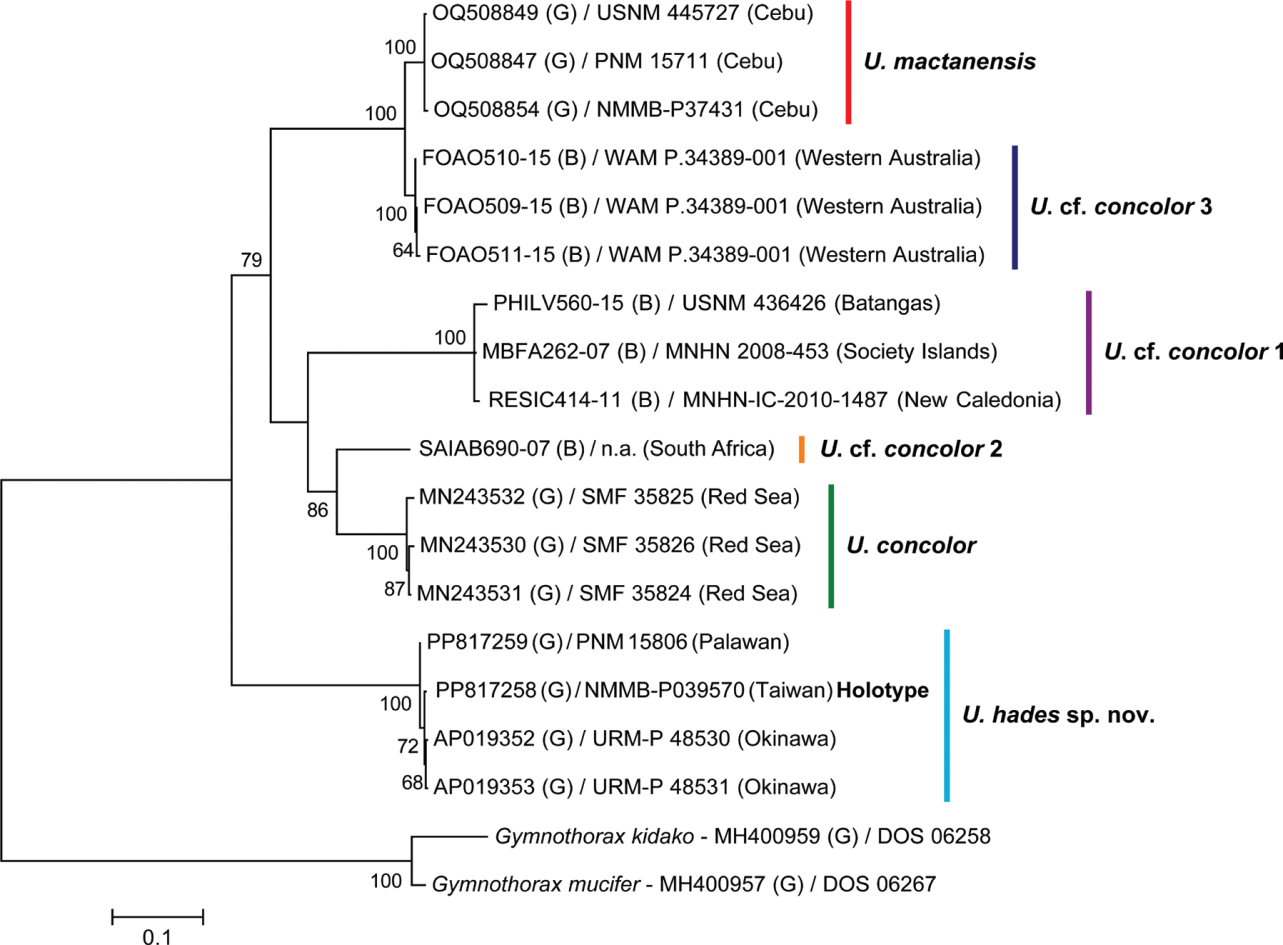
Maximum-likelihood tree of the *Uropterygiusconcolor* species complex based on *COI* sequences (652 bp). *Gymnothoraxkidako* and *G.mucifer* (subfamily Muraeninae) are outgroups. Numerals beside the internal branches are bootstrap values, and values below 60 are not shown. (B) = sequence from BOLD Systems; (G) = sequence from GenBank.

DNA was extracted from muscle tissues of the holotype (NMMB-P039570) and a paratype (PNM 15806) of the new species. A fragment of partial *COI* gene (680 bp) was amplified by polymerase chain reaction (PCR) using the primers FishF2 (5′-TCG ACT AAT CAT AAA GAT ATC GGC AC-3′) and FishR2 (5′-ACT TCA GGG TGA CCG AAG AAT CAG AA-3′) (Ward et al. 2005). Details for PCR thermal cycling conditions, PCR product purification, and DNA sequencing can be found in Huang et al. (2021a). Obtained sequences were manually edited and assembled in MEGA version 11 (Tamura et al. 2021), and the newly generated *COI* sequences were submitted to GenBank (refer to Fig. 2 for their accession numbers).

All available *COI* sequences of *U.concolor* and U.cf.concolor, as well as three sequences of *U.mactanensis*, from the online databases (GenBank and BOLD Systems) were downloaded for molecular comparisons. After aligning and trimming, a length of 652 bp was retained for analyses. A genetic tree of *COI* sequences was reconstructed based on the maximum-likelihood (ML) method conducted in MEGA v. 11. The HKY + *Γ* + *I* substitution model (Hasegawa et al. 1985) was applied, and a bootstrap analysis with 1,000 replicates was conducted (Felsenstein 1985) for tree building. Sequences of *Gymnothoraxkidako* (Temminck & Schlegel, 1846) and *Gymnothoraxmucifer* Snyder, 1904, from the subfamily Muraeninae, were used as outgroups. Genetic distances of *COI* sequences between the new species and *U.concolor*, U.cf.concolor, and *U.mactanensis* were also calculated using the Kimura 2-Parameter (K2P) model (Kimura 1980).

## ﻿Results

### ﻿Taxonomy

#### ﻿ Uropterygius hades sp. nov.

Taxon classificationAnimaliaAnguilliformesMuraenidae

F3796406-C030-55A7-9C70-2BED3A537469

https://zoobank.org/371622EE-A086-4645-8C85-2FC37461497C

**Figure 3.**
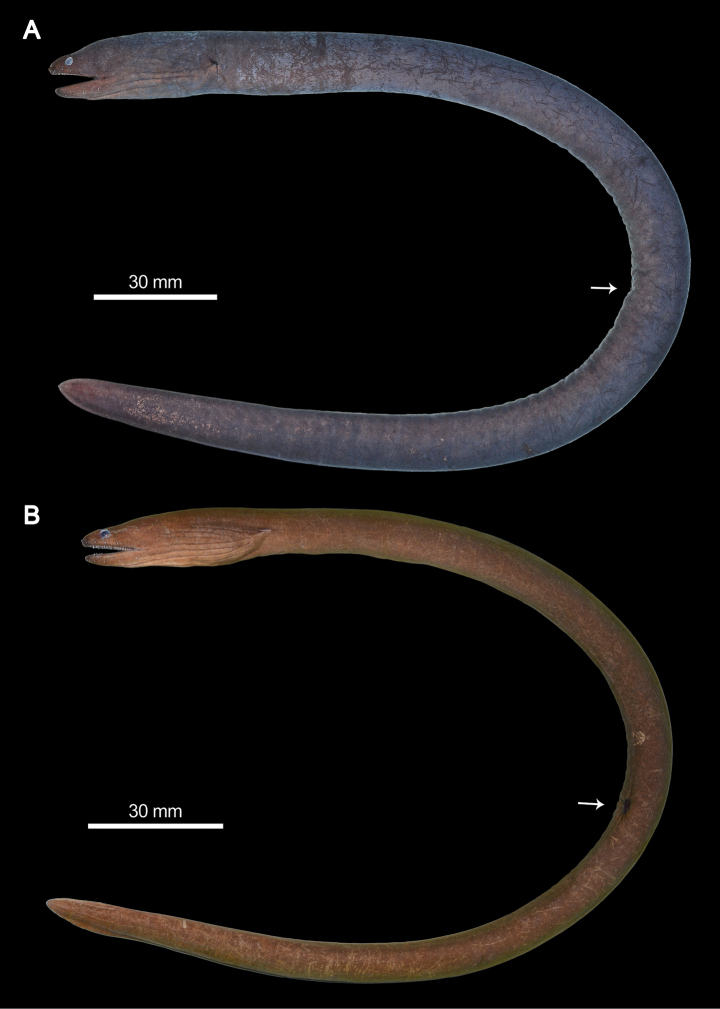
Fresh colorations of *Uropterygiushades* sp. nov. **A** NMMB-P039570, holotype, 349 mm TL, male **B**PNM 15806, paratype, 313 mm TL, female. Arrows indicate the position of the anus.

**Figure 4.**
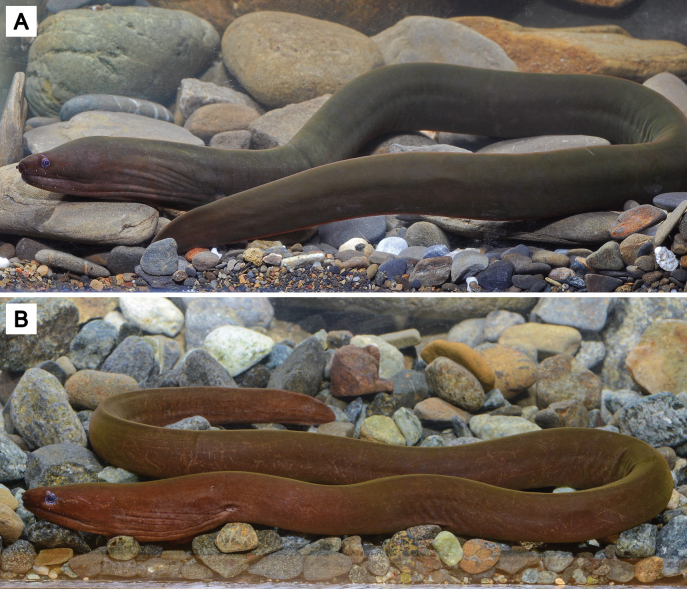
Live photos of *Uropterygiushades* sp. nov. **A** NMMB-P039570, holotype, 349 mm TL, male **B**PNM 15806, paratype, 313 mm TL, female.

**Figure 5.**
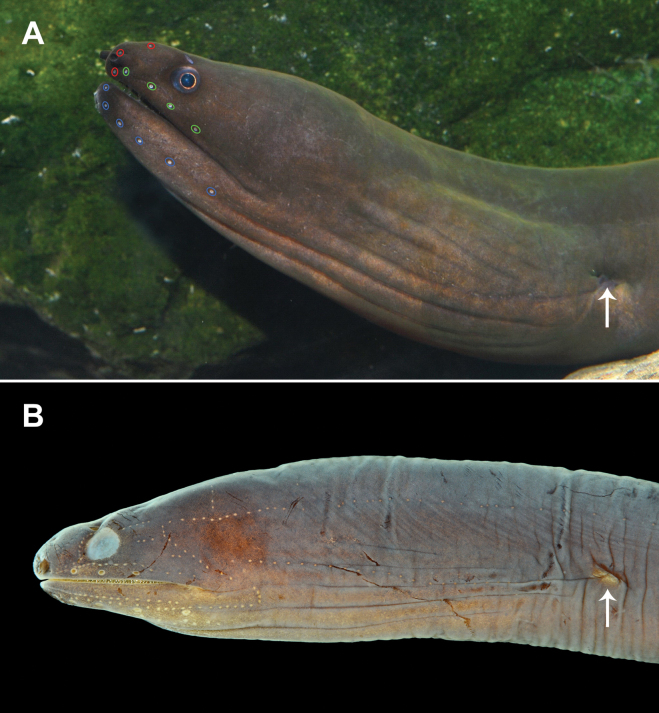
Lateral views of the head of *Uropterygiushades* sp. nov. showing the positions of cephalic sensory pore series and whitish superficial neuromasts **A** NMMB-P039570, holotype, 349 mm TL; red circles for supraorbital pores; green circles for infraorbital pores; blue circles for preoperculo-mandibular pores **B**ZRC 44083, non-type specimen, 148 mm TL. Arrows indicate the gill opening.

**Figure 6.**
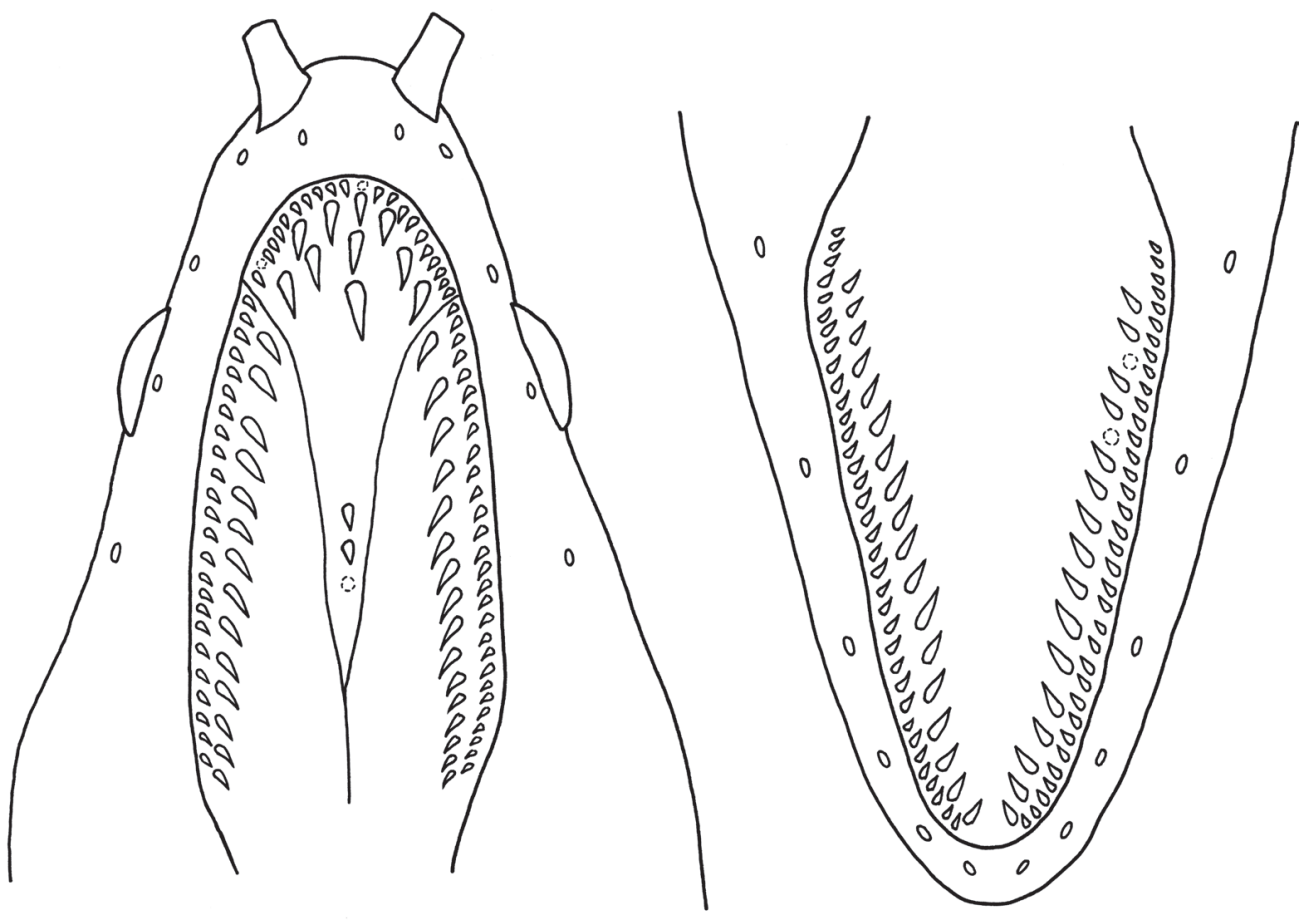
Dentition of *Uropterygiushades* sp. nov., NMMB-P039570, holotype. Upper jaw (left) and lower jaw (right). Dotted circles represent the sockets of missing teeth.

**Figure 7.**
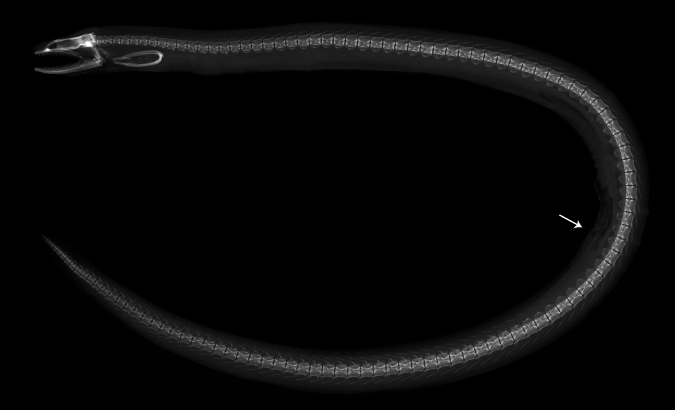
Radiograph showing the skeletal structure of *Uropterygiushades* sp. nov., PNM 15806, paratype, 313 mm TL. Arrow indicates the position of anus.

Figs 3 , 4 , 5 , 6 , 7 , Table 1 Common name: Hades’ snake moray

Uropterygius concolor (not of Rüppell): Sakai and Sato 1982: 79, fig. 1, pl. I-A (Amami and Okinawa islands, Japan); Hatooka and Yoshino 1982: 95, fig. 6, pl. IV (Okinawa and Ishigaki islands, Japan); McCosker et al. 1984: 262 (Amami, Okinawa, and Ishigaki islands, Japan); Maeda and Tachihara 2006: 19, table 1 (Okinawa Island, Japan); Kanda et al. 2009: fig. 3A, table 1 (Ishigaki island, Japan); Hibino 2018 in Motomura et al. 2018: 25, unnumbered fig. (Kakeroma Island, Japan); Miyake et al. 2019: fig. S1c, table 2 (Okinawa Island, Japan).
Uropterygius cf. concolor 4: Huang et al. 2023a: 595, figs 1, 3, tables 2, 3 (Ryukyu Archipelago, Japan; possibly Fiji and southern Java, Indonesia).

##### Type material.

***Holotype*.** • NMMB-P039570 (349 mm TL, male); estuary of the Zhuan River (24°50'24.7"N, 121°49'18.1"E), Yilan County, northeastern Taiwan; dip net at 1.5 m, 11 January 2024, coll. W.C. Jhuang; GenBank *COI* accession number PP817258.

***Paratypes*.** 10 specimens (163–313 mm TL). **Japan**: • KAUM–I. 128986 (205 mm, sex unknown), Sumiyo Bay, Amami Island, Amami group, Kagoshima Prefecture, 20 March 2019, coll. R. Furuhashi • KAUM–I. 132509 (171 mm, mature female), tidal flat of Sumiyo Bay, Amami Island, Amami group, Kagoshima Prefecture, 31 August 2019, coll. R. Furuhashi • KAUM–I. 153507 (264 mm, mature female), mouth of Yakukachi River, Sumiyo, Amami Island, Amami group, Kagoshima Prefecture, 27 April 2002, coll. T. Yonezawa • KAUM–I. 177723 (215 mm, sex unknown) • KAUM–I. 177724 (190 mm, sex unknown), Setouchi, Kakeroma Island, Amami group, Kagoshima Prefecture, 23 November 2022, coll. S. Hashimoto • KMNH VR 100621 (231 mm, mature female), Oura River, Okinawa Island, Okinawa Prefecture, 20 May 2023, coll. K. Takatsuki • KYUM-PI 4637 (246 mm, sex unknown), Oura River, Okinawa Island, Okinawa Prefecture, 6 October 2014, coll. K. Maeda • URM-P 48530 (163 mm, sex unknown) • URM-P 48531 (204 mm, sex unknown), coll. with KYUM-PI 4637, GenBank accession numbers AP019352 and AP019353. **Philippines**: • PNM 15806 (313 mm, mature female), inside the cave, about 50 m from the entrance of the Puerto Princesa Subterranean River (10°11'55.6"N, 118°55'33.7"E), Palawan, tube trap at the bottom of the river, about 9.6 m depth, 03 May 2023, coll. W.C. Huang, R.A. Balisco, and W.C. Jhuang, GenBank *COI* accession number PP817259.

##### Non-type material.

Three specimens (148–158 mm TL). **Fiji**: • AMS I.43866-001 (158 mm, sex unknown), mid Suetabu River, Vanua Levu, February 2006. **Indonesia**: • ZRC 44083 (148 mm, sex unknown), Ujung Genteng, southern Java, obtained through aquarium trade, 02 October 1999. **Philippines**: • ZRC 63518 (155 mm, sex unknown), Matutinao River, Badian, Cebu, 25 November 2001.

##### Diagnosis.

A small, slender moray eel, possible maximum TL <350 mm, female mature at 171 mm TL. Anus at mid-length of body. Eyes small and anteriorly placed. Snout pointed. Upper jaw slightly longer than lower jaw. Teeth sharply pointed with smooth edges and recurved tips; intermaxillary teeth in 5 rows; maxillary and dentary teeth biserial, inner rows extending to about posterior end of jaws; vomerine teeth in single row. No branchial pore. Body uniformly dark brown; head pores, oral cavity, and inner skin of posterior nostril and gill opening whitish; iris reddish-brown. Total vertebrae 117–122.

##### Description.

Values shown below from all the 14 specimens, including holotype, paratypes, and non-types. Proportions in percentage of TL: tail length 47.6–51.4 (*x̄* = 49.6); preanal length 48.6–52.4 (*x̄* = 50.4); trunk length 35.8–41.3 (*x̄* = 38.6); head length 10.5–12.8 (*x̄* = 11.8); body depth at gill opening 2.9–4.4 (*x̄* = 3.7); body depth at anus 3.3–5.0 (*x̄* = 3.8). Proportions in percentage of HL: length of upper jaw 26.3–35.1 (*x̄* = 29.9); length of lower jaw 25.5–33.6 (*x̄* = 28.9); interorbital width 5.9–9.6 (*x̄* = 7.9); snout length 9.3–12.0 (*x̄* = 10.5); eye diameter 5.0–7.2 (*x̄* = 5.8). Vertebral counts: pre-anus vertebrae 55–58 (*x̄* = 57); pre-dorsal fin vertebrae 102–109 (*x̄* = 105); pre-anal fin vertebrae 103–110 (*x̄* = 106); total vertebrae 117–122 (*x̄* = 119) (Table 1).

**Table 1.** Morphometric measurements, teeth, and vertebral counts of *Uropterygiushades* sp. nov. and *U.mactanensis*. Mean values are indicated in parentheses and mode values are indicated in brackets. Abbreviations: HL, head length; TL, total length.

Source	*U.hades* sp. nov.		*U.mactanensis*	
	Holotype	Paratypes & non-types	This study (Iriomote Island)	Huang et al. 2023a
	NMMB-P039570	*n* = 13	*n* = 2	*n* = 21
TL (mm)	349	148–313	316–370	231–342
% TL				
Tail length	47.6	47.9–51.4 (49.8)	51.4–52.5	48.5–52.7 (51.0)
Preanal length	52.4	48.6–52.1 (50.2)	47.5–48.6	47.3–51.5 (49.0)
Trunk length	41.3	35.8–40.3 (38.4)	35.4–35.8	34.5–39.2 (36.6)
Head length	11.2	10.5–12.8 (11.8)	11.7–13.2	11.0–13.5 (12.4)
Body depth at gill opening	4.4	2.9–4.4 (3.6)	5.2–5.6	5.1–6.5 (6.0)
Body depth at anus	3.9	3.3–5.0 (3.8)	4.4–4.9	4.2–6.1 (5.1)
% HL				
Length of upper jaw	35.0	26.3–35.1 (29.5)^a^	33.3–34.9	33.7–40.9 (38.7)
Length of lower jaw	33.6	25.5–33.1 (28.5)^a^	32.7–34.3	33.2–40.8 (38.1)
Snout length	11.7	9.3–12.0 (10.4)	13.8–14.3	12.6–15.7 (14.3)
Interorbital width	9.6	5.9–9.1 (7.8)^a^	9.8–13.0	9.1–12.6 (10.9)
Eye diameter	5.2	5.0–7.2 (5.9)	7.8–9.2	7.8–10.4 (8.8)
Teeth				
Intermaxillary-peripheral	11	8–13 [9]^a^	10–12	9–14
Intermaxillary-intermediate	3–4	3–6 [3]^a^	2	2–5
Intermaxillary-median	3	2–5 [3]^a^	3	2–3
Maxillary-outer	24–28	18–36 [26]^a^	17–22	16–25
Maxillary-inner	14–16	7–19 [11 & 13]^a^	5–6	5–8
Vomerine	3	2–9 [5]^a^	6–8	2–11
Dentary-outer	35–36	26–43 [36]^a^	31–36	27–40
Dentary-inner	18	8–22 [13]^a^	5–7	6–8
Vertebrae				
Pre-anus	58	55–58 (57)^b^	48–50	47–51 (49)
Pre-dorsal fin	105	102–109 (105)^b^	99	95–101 (98)
Pre-anal fin	106	103–110 (106)^b^	102	100–104 (102)
Total	119	117–122 (119)^c^	110–111	107–112 (110)

^a^Data not including AMS I.43866-001. ^b^Data not including AMS I.43866-001 and URM-P 48530. ^c^Data not including URM-P 48530.

A small, slender moray eel, anus at mid-length of body, tail laterally compressed, body depth roughly consistent throughout whole fish except for narrower, pointed head and tail tip (Figs 3, 4). Fins inconspicuous and restricted to posterior portion of tail, caudal fin short. Gill opening small and oval, below lateral midline of body. Eyes small and anteriorly placed, closer to snout tip than to mouth corner, snout/upper jaw length 0.30–0.40 (*x̄* = 0.36). Snout short and somewhat pointed, space between eyes narrow, anterior portion of head triangular in dorsal view. Jaws moderately long, upper jaw slightly longer than lower jaw, teeth not visible when mouth closed. Anterior nostril short and tubular, close to tip of snout, shorter than eye dimeter in length. Posterior nostril a large oval hole with a raised rim, above and posterior to anterior margin of eye, opening upward (Fig. 5).

Three supraorbital pores, first and second pores on tip of snout; first pore below base of anterior nostril; second pore next to upper base of anterior nostril at horizontal level of lower eye margin; third pore on upper margin of snout, above and posterior to first infraorbital pore. Four infraorbital pores, arranged along upper jaw with equal intervals, first pore posteriorly next to base of anterior nostril; second pore below and anterior to eye; third pore below midpoint of eye; fourth pore below and posterior to eye. Six preoperculo-mandibular pores lining along lower jaw anterior to mouth corner (Fig. 5A). No branchial pore observed except in one specimen (ZRC 63518) having one pore on left side of posterior-dorsal head, representing a rare variation.

Teeth sharply pointed with smooth edges and recurved tips. Intermaxillary tooth plate with 5 rows of teeth; peripheral rows with 8–13 (mode 9) tightly arranged small teeth on each side; teeth on intermediate and median rows significantly larger than those on peripheral rows, about twice as tall and depressible, intermediate rows with 3–6 (3) teeth on each side, median row with 2–5 (3) teeth. Maxillary teeth biserial; outer row with 18–36 (26 and 28) teeth, continuous with peripheral intermaxillary teeth of similar size and shape, teeth slightly smaller at posterior end; inner row with 7–19 (11 and 13) straight, widely spaced teeth, continuous with intermediate intermaxillary teeth of approximately the same size and shape, extending to, exceeding, or near posterior end of outer row, with teeth becoming smaller at posterior end. Vomerine with 2–9 (5) small, conical teeth in single row. Dentary teeth biserial; outer row with 26–43 (36) teeth, small and equal-sized, closely arranged; inner row with 8–22 (13) slender and straight teeth, twice taller than teeth on outer row, widely spaced, anterior and posterior teeth smaller than middle ones, extending to or near posterior end of outer row (Fig. 6).

Body uniformly dark brown, color slightly lighter ventrally, covered with greenish mucus when alive. Head pores, oral cavity, and inner skin of posterior nostril and gill opening whitish. Iris reddish-brown. Whitish superficial neuromasts arranged in several lines on head region and in a row along lateral body (Fig. 5B). Preserved color mostly same as in fresh, but slightly faded.

##### Distribution.

This species is widely distributed in estuaries of the Central Indo-Pacific Ocean, ranging from southern Java to Fiji, and extending north to the Ryukyu Archipelago of Japan.

##### Etymology.

The new moray eel is named after Hades, the ancient Greek god of the underworld, in reference to its habitation in turbid estuarine waters, high sensitivity to light, and its uniformly dark coloration, reminiscent of the underworld god. A noun in apposition.

##### Comparisons.

In molecular analyses, the topology of the *COI* tree (Fig. 2) reveals that *U.hades* sp. nov., *U.concolor*, U.cf.concolor 1, U.cf.concolor 2, U.cf.concolor 3, and *U.mactanensis* are monophyletic, which concord with the findings of Huang et al. (2023a). Two *COI* sequences from GenBank, originally identified as “*U.concolor*” (AP019352 and AP019353), clustered with the two *U.hades* sp. nov. sequences generated in this study. The two voucher specimens, URM-P 48530 and URM-P 48531, have been examined and designated as paratypes of *U.hades* sp. nov. Additionally, a sequence from BOLD Systems identified as “*Gymnothoraxaustralicola*” (PHILV560-15) was found to cluster with U.cf.concolor 1 (Fig. 2). This extends the possible distribution range of U.cf.concolor 1 northward to the Philippines, where it overlaps with *U.hades* sp. nov. and *U.mactanensis* (Fig. 1). Lastly, large K2P genetic distances were observed between *U.hades* sp. nov. and *U.concolor* (18.1%), U.cf.concolor 1 (18.5%), U.cf.concolor 2 (17.1%), U.cf.concolor 3 (20.0%), and *U.mactanensis* (19.7%), further supporting the validity of the new species.

In morphological comparisons, *U.hades* sp. nov. can be easily distinguished from *U.concolor* (including its three synonyms) and *U.mactanensis* by its exclusively small eyes (5.0–7.2% vs 7.7–11.0% and 7.8–10.4% of HL), absence of branchial pore (vs one in both species) and extended inner rows of teeth reaching the posterior end of jaws (Table 2). *Uropterygiushades* sp. nov. has a similar vertebral formula to *U.concolor* (Fig. 7), but the former has a shorter tail compared to the latter (47.6–51.4% vs 52.4–60.0% of TL). Additionally, despite overlapping in tail length proportions, *U.hades* sp. nov. differs from *U.mactanensis* by having a narrower body depth at gill opening (2.9–4.4% vs 5.1–6.5% of TL), a shorter snout (9.3–12.0% vs 12.6–15.7% of HL), shorter jaws (25.5–35.1% vs 32.7–40.9% of HL), a narrower interorbital width (5.9–9.6% vs 9.1–13.0% of HL), and more vertebrae (117–122 vs 107–112) (Table 1). Morphological data for the remaining members of the *U.concolor* species complex (i.e., U.cf.concolor 1, U.cf.concolor 2, and U.cf.concolor 3) are quite limited and cannot be compared with the new species, except for one *COI* sequence-bearing specimen of U.cf.concolor 3 (WAM P.34389-001, 301 mm TL) with 112 total vertebrae (Huang et al. 2023a). Nevertheless, the tree topology and genetic distances between each clade strongly support their classification as different species (Fig. 2).

**Table 2.** Comparison of selected characteristics of *Uropterygiushades* sp. nov., *U.concolor*, *U.mactanensis*, and the three synonyms of *U.concolor*: *Anarchiasinsuetus*, *A.vermiformis*, and *Gymnomuraenafusca*.

	Eye diameter (% HL)	Tail length (% TL)	Total vertebrae	*N* of branchial pore	Teeth row extends to the posterior end of jaw?		Source
					Inner maxillary	Inner dentary	
*Uropterygiushades* sp. nov.	5.0–7.2	47.6–51.4	117–122	0	Yes	Yes	This study
*Uropterygiusconcolor*	7.7–11.0	52.4–60.0	117–124	1	Yes	No	1, 2
*Anarchiasinsuetus*	7.9	55.0	113	1	No	No	1, 3
*Anarchiasvermiformis*	7.7	56.5	117	1	n/a	n/a	1
*Gymnomuraenafusca*	10	58.3	114+	1	No	No	1
*Uropterygiusmactanensis*	7.8–10.4	48.5–52.7	107–112	1	No	No	3, this study

1. Böhlke and Smith 2002; 2. Smith et al. 2019; 3. Huang et al. 2023a.

*Uropterygiushades* sp. nov. may also be confused with six uniformly brown moray eels in the genus, namely *Uropterygiuscyamommatus* Huang, Liao & Tan, 2023, *Uropterygiusgenie* Randall & Golani, 1995, *Uropterygiusgolanii* McCosker & Smith, 1997, *Uropterygiusinornatus* Gosline, 1958, *Uropterygiusversutus* Bussing, 1991, and *Uropterygiusxenodontus* McCosker & Smith, 1997. The absence of a branchial pore in *U.hades* sp. nov. can serve as the primary diagnostic characteristic to distinguish it from congeners. *Uropterygiuscyamommatus*, *U.genie*, *U.golanii*, *U.inornatus*, and *U.xenodontus* each possess a single branchial pore, while *U.versutus* has two branchial pores. *Uropterygiushades* sp. nov. also has fewer vertebrae (117–122 total vertebrae) compared to *U.cyamommatus* (141–149), *U.golanii* (145–148), *U.versutus* (131–138), and *U.xenodontus* (152–157), but overlaps with *U.genie* (121–122) and *U.inornatus* (116–133). However, *U.hades* sp. nov. differs from *U.genie* and *U.inornatus* by having smaller eyes (5.0–7.2% vs 10.4–11.4% and 7.7–10.0% of HL), a shorter tail (47.6–51.4% vs 53.5–54.5% and 52.4–54.5% of TL), and different dentition (biserial maxillary teeth vs about 4 rows and uniserial). Refer to table 2 in Huang et al. (2023b) and the references cited therein for more detailed comparisons.

##### Note on additional records of *U.mactanensis*.

**Figure 8.**
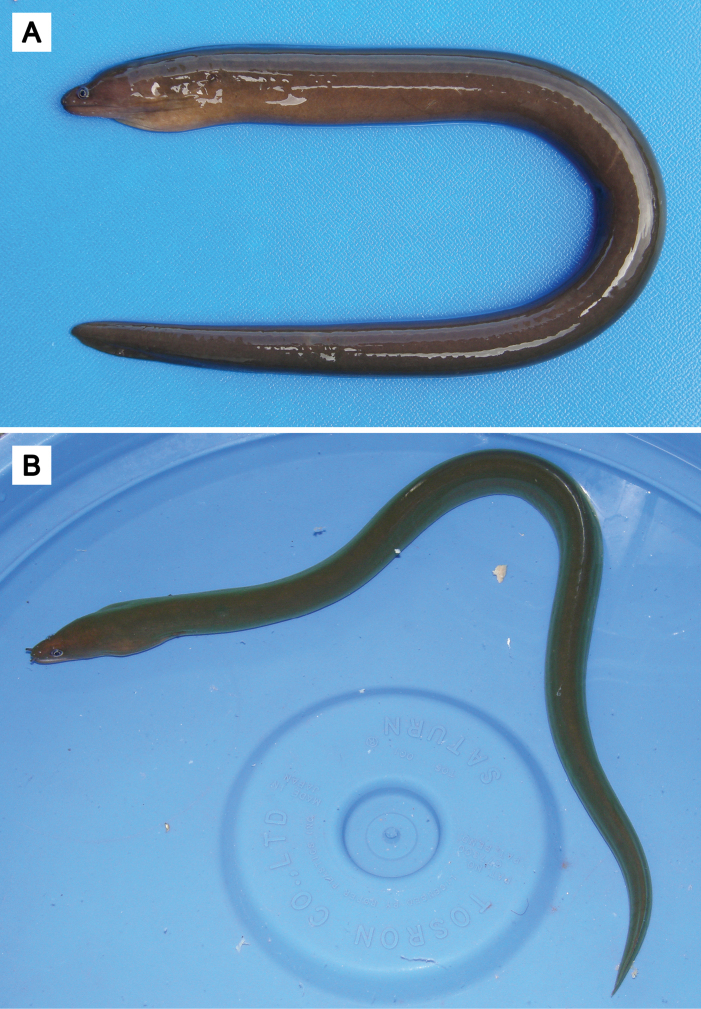
*Uropterygiusmactanensis* collected from Iriomote Island, KYUM-PI 2591, 316 mm TL**A** fresh coloration **B** live photo. Photographed by Atsushi Tawa.

During our survey at several Japanese museums, two specimens having one branchial pore on both sides of head, formerly identified as *U.concolor*, were found from the Kyushu University Museum (catalog numbers KYUM-PI 2591 and 2612) (Fig. 8). Although we were unable to assess the genetic features of these specimens, they can be identified as *U.mactanensis* based on diagnostic characteristics (Table 1). They were collected from the shallow bottoms around two adjacent small reef crests, one located off Funaura and the other off the mouth of the Kura River on Iriomote Island. The collection sites feature coarse sandy areas with large patch reefs, while the shallower regions contain scattered small patches of seagrass. Several marine muraenid species were collected by the same series of tube traps, such as *Echidnanebulosa* (Ahl, 1789), *Gymnothoraxflavimarginatus* (Rüppell, 1830), *Gymnothoraxjavanicus* (Bleeker, 1859), *Gymnothoraxthyrsoideus* (Richardson, 1845), *Gymnothoraxzonipectis* Seale, 1906, and *Scuticariatigrina* (Lesson, 1828).

The second author has examined most museum collections of “*U.concolor*” from Japanese waters, identifying the majority as *U.hades* sp. nov., with only two specimens identified as *U.mactanensis*. This implies the rarity of *U.mactanensis* in the Ryukyu Archipelago. As a rare case of confusion between the two species, the general information previously provided for Japanese “*U.concolor*” (e.g., Tachihara 2017) possibly refers solely to *U.hades* sp. nov. Many photos of “*U.concolor*” collected from the estuary of Iriomote Island can further support the identification of *U.hades* sp. nov. based on general appearance and the absence of branchial pores. These photos can be found on FishPix (https://fishpix.kahaku.go.jp/fishimage-e/) (KPM-NR 275, 3350, 20306, 42937, 42948, 42949, 42950), photographed by H. Senou and T. Suzuki.

##### Material examined.

• KYUM-PI 2591 (316 mm TL), off mouth of Kura River, Iriomote Island, Yaeyama Group, Ryukyu Islands, 1 November 2009, coll. A. Tawa • KYUM-PI 2612 (370 mm TL), off Funaura, Iriomote Island, Yaeyama Group, Ryukyu Islands, 5 November 2009, coll. A. Tawa.

## ﻿Discussion

The absence of branchial pores is an important characteristic of *Uropterygiushades* sp. nov. Although one examined specimen has a branchial pore on the left side of the head, no other morphological differences were found. Intraspecific variations in head pore number are common in moray eels. For example, in the genus *Strophidon* McClelland, 1844, a fourth infraorbital pore is a diagnostic characteristic of *Strophidontetraporus* Huang & Liao in Huang et al. 2020, distinguishing it from congeners with only three pores. Despite this, a few specimens of *Strophidondorsalis* (Seale, 1917), *Strophidonsathete* (Hamilton, 1822), and *Strophidonui* Tanaka, 1918 were observed with a fourth infraorbital pore on only one side of the head (Huang et al. 2020). Similarly, the additional branchial pore in *U.hades* sp. nov. should be considered a rare intraspecific variation and does not affect the general diagnostics of the taxon.

The salinity at the type locality in the Zhuan River was promptly measured using a refractometer, a day after its collection during a spring tide. Measurements showed a salinity of 5‰ during low tide and 19‰ during high tide. Similarly, surface water salinity at the sampling site in Puerto Princesa Subterranean River (PPSR) was measured at 9‰ during the high tide. These findings support that *U.hades* sp. nov. is one of the rare cases of moray eels inhabiting brackish-water environments. Another estuarine moray, *Echidnarhodochilus* Bleeker, 1863, was observed co-occurring with *U.hades* sp. nov. in Japan, Taiwan, and the Philippines (Masuda and Kobayashi 1994; Huang et al. 2021b; Wada and Hibino 2024; W.C. Huang pers. obs.).

**Figure 9.**
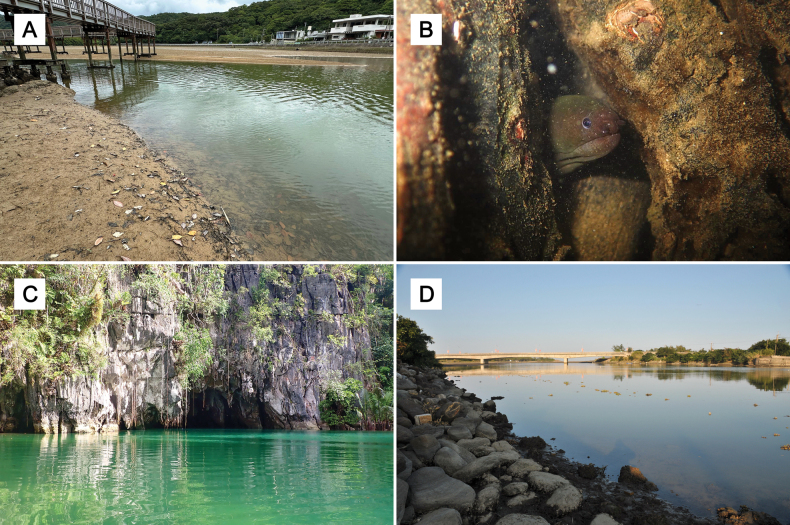
Different habitats of *Uropterygiushades* sp. nov. **A** Oura River, Okinawa Island, photographed by Koki Takatsuki **B** Live photo of a *U.hades* sp. nov. hiding in gaps among mangrove aerial roots, taken at night at the edge of a mangrove forest zone along the Oura River, Okinawa Island, photographed by Hirozumi Kobayashi **C** Puerto Princesa Subterranean River, Palawan, photographed by Wei-Cheng Jhuang **D** Zhuan River, northeastern Taiwan, photographed by Shan-Yu Yang.

*Uropterygiushades* sp. nov. seems to be closely associated with mangroves, as most of its known habitats in the Ryukyu Archipelago are mangrove swamps. The habitat on Okinawa Island is an estuary with many fallen leaves from mangrove trees (Fig. 9A). However, *U.hades* sp. nov. has never been discovered inside the mangrove forest. Instead, it rests in gaps among mangrove aerial roots and leaves (Fig. 9B). When there are fewer fallen leaves, it uses scattered stones as an alternative for concealment. On Amami Island, one collection site is far outside the river mouth and lacks mangrove trees. It consists of a muddy bottom with many gravels and stones, with a tiny freshwater seepage from the land side, similar to the environment of Kakeroma Island. For Philippine specimens, although *U.hades* sp. nov. was collected inside the PPSR, the entrance of the cave also harbors a few mangrove trees (Fig. 9C), analogous to the estuarine environment of the Matutinao River in Cebu. The Zhuan River, where the holotype was discovered, emerges as another known habitat of *U.hades* sp. nov. without mangrove trees (Fig. 9D). These observations imply that while mangroves may be important for the survival of *U.hades* sp. nov., brackish water is a more essential factor.

One possible interpretation for the preference of *U.hades* sp. nov. to mangroves as habitat is that mangroves can facilitate the accumulation of fine sediment, thereby creating a soft-mud substrate that may be suitable for *U.hades* sp. nov. (Perry and Berkeley 2009; Tachihara 2017). We observed that *U.hades* sp. nov. exhibits tail-first burrowing behavior when kept in an aquatic tank, similar to snake eels (family Ophichthidae) which typically inhabit muddy or sandy substrates. Additionally, the reduction in the number of head pores is hypothesized to help avoid clogging by the substrate, as this phenomenon is observed in certain eel species that inhabit sand and mud burrows (McCosker 1977; McCosker and Randall 2007). Although there are no mangroves directly adjacent to the sampling sites at the Zhuan River and the PPSR, both locations feature muddy, silty bottoms with rocks, similar to the substrates found in mangrove swamps.

Furthermore, we observed that *U.hades* sp. nov. is highly sensitive to light and consistently attempts to hide when exposed to it. This suggests that it may typically inhabit turbid waters such as estuaries, resulting in its lack of acclimation to light exposure. The small eye proportion of *U.hades* sp. nov. may also indicate its adaptation to low-light conditions, wherein they primarily use their chemoreception rather than vision to detect prey or avoid predators. A reduction in eye size is also observed in some congeners, such as *U.cyamommatus* (eye diameter 3.0–4.6% of HL) and *Uropterygiusoligospondylus* Chen, Randall & Loh in Loh et al. 2008 (eye diameter 3.9–7.1% of HL). The former is found in anchialine caves, whereas the latter inhabits intertidal zones consisting of boulders frequently hit by strong waves. Both environments pose challenges to visual perception, making reliance on other senses more important (Loh et al. 2008; Hibino et al. 2020; Koreeda et al. 2020; Huang et al. 2023b).

Combining information from habitat type, body structure, and behavior, we propose that *U.hades* sp. nov. is an estuarine moray eel that inhabits turbid waters with muddy and soft substrates, using its tail to burrow and hide in sediments, among rocks, or in fallen mangrove leaves. While this study addresses a portion of the *U.concolor* species complex conundrum, the diversity of these small, uniformly brown moray eels may still be underestimated. For instance, morphological and genetic data reveal the presence of at least three sympatric species found in the Philippines (i.e., *U.hades* sp. nov., *U.mactanensis*, and U.cf.concolor 1), while both *U.hades* sp. nov. and *U.mactanensis* can be found at Iriomote Island. This high diversity could be triggered by niche segregation and adaptation to different environments among species; for example, *U.concolor* inhabits shallow fringing reefs, *U.mactanensis* prefers reef-seagrass interfaces, and *U.hades* sp. nov. thrives in muddy estuaries (Smith et al. 2019; Huang et al. 2023a). Additionally, a phylogenetic analysis reveals the non-monophyly of the *U.concolor* species complex, indicating that these morphologically similar moray eels have multiple evolutionary origins (Smith et al. 2019). Further studies are needed to clarify their taxonomic status, as three synonyms of *U.concolor* and at least three unresolved genetic lineages persist within the species complex.

## Supplementary Material

XML Treatment for Uropterygius hades

## ﻿Additional information

### Author ORCIDs

Wen-Chien Huang https://orcid.org/0000-0002-9482-8188

Yusuke Hibino https://orcid.org/0000-0002-5670-3851

Rodulf Anthony Balisco https://orcid.org/0000-0001-9503-0912

Te-Yu Liao https://orcid.org/0000-0001-7588-200X

### Data availability

All of the data that support the findings of this study are available in the main text.
